# Prognostic significance of peripheral lymphocyte counts in Parkinson’s disease

**DOI:** 10.1016/j.prdoa.2025.100344

**Published:** 2025-05-10

**Authors:** Shinsuke Omata, Hiroaki Fujita, Hirotaka Sakuramoto, Keitaro Ogaki, Keisuke Suzuki

**Affiliations:** Department of Neurology, Dokkyo Medical University, Japan

**Keywords:** Parkinson’s disease, Inflammation, Lymphocyte, Disease outcome

## Abstract

**Background:**

Inflammation plays a pivotal role in the pathogenesis of Parkinson’s disease (PD). The aim of this study was to explore whether peripheral blood markers are associated with subsequent disease outcomes in patients with PD.

**Methods:**

We conducted a follow-up study among consecutive patients with PD and investigated the association between outcomes and baseline blood markers, including neutrophil, lymphocyte, and monocyte counts; the neutrophil-to-lymphocyte ratio; motor/nonmotor symptoms; and radionuclide scanning findings.

**Results:**

Of the 112 patients, 97 (87%) met the eligible criteria. Over the average 1800-day follow-up period, 33 patients completed the full follow-up, 30 patients reached an endpoint (death, 3; hospitalization, 12; and placement in a nursing home, 6; home medical care 9), and 34 patients were censored. At the baseline assessment, patients who reached an endpoint were older and had higher Hoehn and Yahr stages and motor symptom scores, larger reductions in cardiac metaiodobenzylguanidine scintigraphy accumulation and lower peripheral lymphocyte counts than patients who completed follow-up or were censored. Kaplan–Meier curve analysis revealed that patients who had low peripheral lymphocyte counts had a shorter time to reach an endpoint than those with preserved lymphocyte counts. According to the multivariate Cox proportional hazards regression analysis, motor symptoms and peripheral lymphocyte count were associated with reaching an endpoint.

**Conclusions:**

Among the blood biomarkers, a lower lymphocyte count was associated with worse outcomes in patients with PD. Low peripheral lymphocyte counts may be predictive of subsequent worse PD outcomes.

## Highlights

• PD patients who reached an endpoint had lower lymphocyte counts at baseline.
• PD patients with low lymphocyte counts reached an endpoint earlier.
• Motor symptoms and lymphocyte count were associated with reaching an endpoint.

## 1 Introduction

Parkinson’s disease (PD) is a common neurodegenerative disorder that leads to major disability and an increasing global public health burden owing to its motor, nonmotor, and cognitive features. Although precise mechanisms causing widespread neurodegeneration remain unclear, a growing body of evidence suggests that neuroinflammation, including microglia activation, infiltration of lymphocytes and elevated inflammatory cytokines, plays a critical role in the pathogenesis of PD [1,2]. Immune dysregulation is not limited to the brain of PD patients, as circulating immune cells have also been shown to be impaired, with CD4+ T lymphocytes playing an important role [1,[3], [4], [5]]. In a study of postmortem human PD specimens and a 1-methyl-4-phenyl-1,2,3,6-tetrahydropyridine (MPTP) mouse model of PD, CD4+ and CD8 + T cell infiltration was observed, and MPTP-induced nigrostriatal dopaminergic cell death was attenuated by the removal of CD4+ T cells [6]. An in vitro study of peripheral blood mononuclear cells from patients with PD revealed that patient-derived T cells (mostly CD4+ T cells) specifically recognized α-synuclein peptides [7]. The role of α-synuclein in the activation of microglia and neurotoxic responses by CD4+ T cells has been suggested [3]; CD4+ regulatory T cells seem to induce microglia apoptosis, and this maladaptive immune and inflammatory response may be associated with the pathogenesis of PD [1,8]. Conversely, peripheral inflammation, likely triggered by gut microbiota, may accelerate α-synuclein aggregation and deposition [8]. Peripheral blood neutrophil, lymphocyte, and monocyte counts are easy-to-obtain biomarkers that reflect the degree of peripheral inflammation. Several studies have consistently reported that patients with PD present higher neutrophil counts, neutrophil-to-lymphocyte ratio (NLR) and monocyte counts and lower lymphocyte counts than healthy controls [[8], [9], [10], [11], [12], [13], [14], [15]]. The decrease in the number of circulating lymphocytes was primarily due to a decrease in circulating CD4+ T cells, accompanied by subset imbalances and functional dysregulation, particularly a bias towards pro-inflammatory phenotypes, such as T helper (Th) 1 and Th17 cell subsets [3,4,8]. The lower lymphocyte count might be causally related to the subsequent development of PD [16], which may be related to a compromised blood–brain barrier (BBB) in the vicinity of the midbrain and increased T cells infiltrating affected brain regions [17]. Several studies have suggested the possibility that peripheral inflammation is associated with the clinical presentation of PD, such as comorbidity of rapid eye movement sleep behavioral disorder (RBD), cognitive decline, olfactory loss, cerebrospinal fluid findings, motor subtypes and rapid disease progression [4,[11], [12], [13], [14], [15],[18], [19], [20], [21], [22]]. We hypothesized that peripheral inflammation, as assessed by routine peripheral blood tests, would predict worsening PD progression and may result in the discontinuation of regular hospital visits. Here, we conducted a retrospective study to examine the associations between peripheral blood markers and the subsequent PD course.

## 2 Methods

This single-center, cross-sectional study was performed in accordance with the principles of the Declaration of Helsinki and approved by the Institutional Review Board of Dokkyo Medical University. All participants provided written informed consent.

## 3 Subjects

### 3.1 Inclusion criteria

The consecutive patients with PD who were newly diagnosed or referred to our hospital and who had a following comprehensive assessment in the initial evaluation period from April 2018 to March 2020 were potential candidates. The diagnosis of PD was confirmed on the basis of the published clinical diagnostic criteria of the International Parkinson and Movement Disorder Society [23].

### 3.2 Exclusion criteria

Patients with a history of cerebrovascular disease according to medical records or brain magnetic resonance imaging or with a changed diagnosis of PD during the follow-up period were excluded. Bedridden patients and patients with dementia (defined as having a Mini-Mental State Examination (MMSE) score less than 20) who were unable to complete the questionnaire were excluded. Patients were also excluded if they had a history of cancer or hematological malignancy; had an active or chronic autoimmune, inflammatory, or infectious disease; or were receiving immunosuppressive therapy.

### 3.3 Initial assessment

Between April 2018 and March 2020, all participants underwent the following assessments. Disease severity was rated using the Hoehn and Yahr (HY) staging. Motor symptoms were evaluated with the Movement Disorder Society Unified Parkinson’s Disease Rating Scale (MDS-UPDRS) Part III. Cognitive function was assessed with the Japanese version of the MMSE. Levodopa equivalent doses (LEDs) were calculated via previously described methods [24]. Olfactory function was examined with Open Essence (Wako, Japan), a card-type odor identification test [25]. PD-related sleep symptoms, daytime sleepiness, and RBD symptoms were assessed with the Japanese version of the Parkinson’s Disease Sleep Scale-2 (PDSS-2), Epworth Sleepiness Scale (ESS) and RBD Screening Questionnaire (RBDSQ-J) [26]. Autonomic symptoms were assessed using the Japanese version of the Scale for Outcomes in Parkinson’s Disease–Autonomic (SCOPA-AUT) [27]. Depressive symptoms were assessed with the Beck Depression Inventory-II (BDI-II). Cardiac metaiodobenzylguanidine (MIBG) scintigraphy findings indicating postganglionic cardiac sympathetic denervation were included in the MDS supportive criteria for PD [23]. The subjects were instructed to remain in a supine position for 15 min, while 111 MBq ^123^I-MIBG (Fujifilm RI Pharma Co., Tokyo, Japan) was intravenously injected. Chest single-photon emission computed tomography (SPECT) and planar images were obtained using a gamma camera fifteen minutes (early phase) and four hours (delayed phase) after the injection. The heart-to-mediastinum ratio (H/M ratio) was calculated by dividing the count density of the left ventricular region of interest (ROI) by that of the mediastinal ROI, as described previously [28]. In the present study, the H/M ratios in the early and delayed phases were analyzed. ^123^I FP-CIT-SPECT (dopamine transporter single-photon emission computed tomography, DAT-SPECT) imaging was performed 3 h after an injection of 167 MBq (4.5 mCi). The specific binding ratio (SBR) of the striatum was semiquantitatively determined and analyzed using the QSPECT DAT quantification software program (Molecular Imaging Laboratory, Inc., Osaka, Japan) [29]. The lower side of the SBR was used for the analysis. The blood sampling was performed after overnight fasting, and the average of three consecutive blood draws during the initial evaluation period was used. The counts of neutrophils, lymphocytes, and monocytes in blood samples were assessed. The NLR was calculated as the absolute neutrophil count (×10^3^ cells/μL) divided by the absolute lymphocyte count (×10^3^ cells/µL). The participants were divided into two groups on the basis of the median neutrophil, lymphocyte and monocyte counts and NLR. Afterward, all patients visited our hospital at one- to two-month intervals for regular follow-up with a movement disorder specialist.

### 3.4 Endpoints

In March 2024, we carefully checked the medical records from the initial assessment of each patient. Patients who were referred to other facilities for reasons unrelated to their PD conditions, such as relocation or the development of other diseases, or who self-interrupted their visits were counted as censored patients. We considered all PD-related events that resulted in the discontinuation of follow-up as an endpoint. In our cohort, death, hospitalization, nursing home admission, and transition to home health care were included as endpoints. Hospitalization was defined as a hospital stay of one week or longer and did not include hospitalizations only for PD-medication adjustment. Placement in a nursing home was defined as a stay in a nursing home for at least two weeks regardless of the level of care. Patients who were still being followed up at our hospital in March 2024 were considered to have completed follow-up.

### 3.5 Statistical analyses

After the final follow-up in March 2024, the outcome of each patient was evaluated and classified as follows: completed follow-up, reached an endpoint, and censored. Additionally, clinical backgrounds at baseline were compared retrospectively. To compare overall differences in continuous variables, the Kruskal–Wallis test was used, as appropriate. Similarly, the chi-square test was used to compare categorical variables. For post hoc multiple comparisons, the Mann–Whitney *U* test with Bonferroni correction was performed. We then divided patients into two groups by either a low or preserved number of lymphocytes and compared clinical variables, using the Mann–Whitney *U* test or the chi-square test, as appropriate. Kaplan–Meier survival curves with the log-rank test were used to assess the time from the initial assessment to reach an endpoint and to compare the clinical course according to high and low blood marker levels. Multivariate Cox proportional hazards regression analysis was performed to calculate hazard ratios (HRs) with 95 % confidence intervals (CIs) for the effects of blood markers on reaching an endpoint, adjusting for age, sex, disease duration, MDS-UPDRS Part III, and LEDs (adjusted model). A two-tailed P value < 0.05 was considered to indicate statistical significance. IBM SPSS Statistics version 29 (IBM SPSS, Tokyo, Japan) was used for the statistical analyses. The sample size was calculated using a Power and sample size program [30]. We planned the study with an accrual interval of 730 days and an additional follow-up after the accrual interval of 1460 days. Based on prior studies [19,21,22], we assumed the median time to endpoint in PD for high- and low-lymphocyte patients is 1460 and 700 days, respectively, and we needed 49 PD with low lymphocyte patients and 49 PD with control subjects to be able to reject the null hypothesis, with equal experimental and control survival curves with a power of 0.8. The Type I error probability associated with this test of this null hypothesis is 0.05.

## 4 Results

**Fig. 1:**
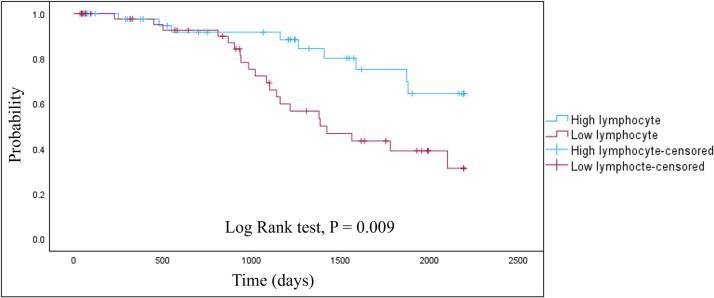
Kaplan–Meier curve for reaching an endpoint in the group with preserved lymphocyte counts (n = 48) and the group with low lymphocyte counts (n = 49).

During the initial evaluation periods, 112 patients underwent the comprehensive assessment, and 97 patients were included after excluding patients who met the exclusion criteria (Supplementary Fig. 1). The mean age of the participants was 69.2 years, and 49 participants were women. The mean disease duration was 3.3 years. Over the average 1800-day follow-up period, 33 patients completed the full follow-up, 30 patients reached an endpoint (death, 3; hospitalization, 12; nursing home admission, 6; home medical care, 9), and 34 patients were censored (referrals to clinics near home, 22; self-interruption, 6; hospitalization for other diseases, 3; and relocation, 3). The clinical backgrounds of all the participants and each group based on outcomes are shown in Table 1. The patients who reached an endpoint were older and had a high HY stage and MDS-UPDRS Part III score, larger reductions in cardiac MIBG scintigraphy accumulation and lower peripheral lymphocyte counts than patients who completed follow-up or were censored. There were no significant differences in other nonmotor symptoms, LEDs, or peripheral blood neutrophil and monocyte counts and NLR. Then we compared the clinical variables between groups divided by the number of lymphocytes. The lower lymphocyte group was older and had higher NLR and lower monocyte counts than the group with preserved lymphocyte counts (Supplementary Table 1). Kaplan–Meier curve analysis revealed that patients who had low peripheral lymphocyte counts had a shorter time to reach an endpoint than did those with preserved lymphocyte counts (Fig. 1, log rank test, P = 0.009). There were no statistically significant differences in the clinical course when groups were divided based on the peripheral neutrophil count, monocyte count or the NLR (Supplementary Fig. 2). According to the Cox proportional hazards regression analysis, higher age, higher HY stage, higher MDS-UPDRS Part III score, lower SBR value on DAT-SPECT, and lower lymphocyte count were associated with reaching an endpoint in the unadjusted model (Table 2). The multivariate Cox model revealed higher MDS-UPDRS part III score and lower peripheral lymphocyte count were associated with reaching an endpoint in the adjusted model (adjusting for age, sex, disease duration, and LEDs).

**Table 1:** The clinical backgrounds of all the participants and each group on the basis of outcomes.

	**All PD patients (n = 97)**	**Completed follow-up (n = 33)**	**Lost to follow-up** **(n = 34)**	**Reached endpoint (n = 30)**	**P value**
**Age (years)**	69.2 ± 8.7	66.5 ± 8.9*	68.0 ± 8.6^¶^	73.4 ± 7.1*^¶^	**0.004**
**Sex (M/F)**	48/49	19/14	13/21	16/14	0.251
**De novo (%)**	28 (28.9)	5 (15.2)	13 (38.2)	10 (33.3)	0.092
**PD duration (years)**	3.3 ± 3.4	3.4 ± 2.9	3.0 ± 3.9	3.6 ± 3.4	0.136
**MMSE score**	24.7 ± 6.6	25.7 ± 5.5	24.3 ± 8.4	24.2 ± 5.3	0.109
**HY stage**	2.6 ± 1.0	2.7 ± 0.9	2.3 ± 0.8^¶^	3.0 ± 1.1^¶^	**0.031**
**MDS-UPDRS III score**	31.7 ± 15.8	31.9 ± 17.7	27.3 ± 14.9^¶^	37.3 ± 13.6^¶^	**0.047**
**LEDs (mg/day)**	324.8 ± 324.4	352.7 ± 325.7	257.0 ± 286.4	371.0 ± 360.0	0.217
**SCOPA-AUT**	10.9 ± 7.8	9.9 ± 6.8	10.6 ± 6.7	12.5 ± 9.8	0.676
**DAT-SPECT SBR (low)**	3.2 ± 1.1	3.3 ± 1.3	3.4 ± 1.0	2.8 ± 0.8	0.119
**MIBG H/M** **(early)**	2.3 ± 2.6	2.8 ± 4.5	2.2 ± 0.6^¶^	1.8 ± 0.4^¶^	**0.046**
**MIBG H/M** **(delay)**	2.0 ± 2.5	2.5 ± 4.2	2.0 ± 0.9^¶^	1.6 ± 1.0^¶^	**0.015**
**Olfactory test score**	3.7 ± 2.3	3.8 ± 1.9	4.2 ± 2.2	2.9 ± 2.6	0.052
**PDSS-2 score**	12.8 ± 8.8	12.4 ± 8.8	13.0 ± 7.8	12.9 ± 10.1	0.741
**ESS score**	7.5 ± 5.8	6.8 ± 5.9	7.8 ± 5.8	8.0 ± 5.9	0.695
**RBDSQ-J score**	2.7 ± 4.0	2.9 ± 3.5	1.7 ± 1.7	3.5 ± 0.9	0.425
**BDI-II score**	13.2 ± 8.8	11.3 ± 7.8	14.2 ± 8.3	14.1 ± 10.4	0.294
**Neutrophil count (×10^3^/μL)**	3.82 ± 0.99	3.73 ± 0.72	4.17 ± 1.18	3.53 ± 0.93	0.094
**Lymphocyte count** **(×10^3^/μL)**	1.48 ± 0.53	1.61 ± 0.45*	1.63 ± 0.61^¶^	1.19 ± 0.38*^¶^	**<0.001**
**NLR**	2.94 ± 1.49	2.47 ± 0.76	2.95 ± 1.43	3.44 ± 1.99	0.098
**Monocyte count** **(×10^3^/μL)**	0.338 ± 0.118	0.339 ± 0.112	0.362 ± 0.144	0.310 ± 0.088	0.419

The data are presented as the n (%) or means ± standard deviations (SDs). Statistically significant (P < 0.05) overall differences among the three groups are shown in bold.

*^¶^ P < 0.05 in multiple comparisons performed using the Mann–Whitney *U* test and Bonferroni correction. The same symbols indicate items with significant differences.

PD, Parkinson’s disease; MMSE, Mini-Mental State Examination; HY, Hoehn and Yahr; MDS-UPDRS, Movement Disorder Society Unified Parkinson’s Disease Rating Scale; LEDs, levodopa equivalent doses; SCOPA-AUT, Scales for Outcomes in Parkinson’s Disease–Autonomic; DAT-SPECT, dopamine transporter single-photon emission computed tomography; SBR, specific binding ratio; MIBG, metaiodobenzylguanidine; H/M ratio, heart–to-mediastinum ratio; NLR, neutrophil–lymphocyte ratio.

**Table 2:** Cox regression model for the associations between clinical parameters and reaching an endpoint.

**Unadjusted**			**Adjusted**	
	**HR**	**P value**	**HR**	**P value**
**Age**	1.086 (1.034–1.140)	**<0.001**	1.052 (0.997–1.111)	0.066
**Sex (Ref: male)**	0.907 (0.442–1.860)	0.789	0.847 (0.377–1.900)	0.687
**Disease duration**	0.987 (0.890–1.095)	0.806	1.013 (0.893–1.150)	0.839
**HY stage**	1.462 (1.033–2.068)	**0.032**	−	
**MDS-UPDRS Part III**	1.029 (1.004–1.053)	**0.020**	1.028 (1.001–1.055)	**0.039**
**DAT SPECT, low SBR**	0.594 (0.397–0.889)	**0.011**	−	
**MIBG H/M ratio delay**	0.847 (0.518–1.386)	0.509	−	
**LEDs**	1.000 (0.999–1.001)	0.874	1.001 (0.999–1.003)	0.226
**Neutrophil count**	0.641 (0.434–1.134)	0.702	−	−
**Lymphocyte count**	0.242 (0.104–0.561)	**<0.001**	0.318 (0.105–0.964)	**0.043**
**NLR**	1.154 (0.971–1.367)	0.104	−	−
**Monocyte count**	0.122 (0.004–3.733)	0.228	−	−

The adjusted model included age, sex, disease duration, MDS-UPDRS Part III, LEDs, and lymphocyte count.

Statistically significant values (P < 0.05) are shown in bold.

HR; hazard ratios; HY, Hoehn and Yahr; DAT-SPECT, dopamine transporter single-photon emission computed tomography; SBR, specific binding ratio; MIBG, metaiodobenzylguanidine; H/M ratio, heart-to-mediastinum ratio; LEDs, levodopa equivalent doses; NLR, neutrophil–lymphocyte ratio.

## 5 Discussion

Our study showed that the progression of PD was associated with peripheral blood markers but only with lymphocyte counts. The peripheral blood cell markers have been noted to be associated with various clinical features of PD, with heterogeneous results [4,11,12,14,[18], [19], [20]]. The different peripheral biomarker findings between sporadic and genetic patients with PD was reported [13], suggesting that genetic diversity may be one reason for the discrepancy in the results. The comorbidity of RBD in patients with PD has shown higher inflammatory findings than PD patients without RBD [15], and potential RBD comorbidity may also contribute to the differences in blood marker results. In contrast, the RBD symptom score was not significantly different among groups divided by outcomes in the present study.

The precise mechanism by which lymphocyte depletion affects the progression in PD is unknown. In addition to a decrease in circulating CD4+ T cells, an imbalance in peripheral CD4+ T cell subsets, particularly a bias toward inflammatory Th1 and Th17 cell subsets, has been reported in PD [3,4,8]. In PD, naive CD4+ T cells differentiate into Th1 lineages and increase production of IFN-γ and TNF-α [5]. The decreased production of IL-10 by CD4+ effector T cells likely contributes to the amplification of the Th1 bias [1,5]. Higher pro-inflammatory cytokines, such as IFN-γ, TNF-α, and IL-6, and lower anti-inflammatory cytokines, such as IL-4 and IL-13, were associated with more rapid motor progression in PD [19]. In two studies using the Parkinson’s Progression Marker initiative (PPMI) cohort, a lower baseline lymphocyte count was associated with cognitive decline in PD patients with the apolipoprotein E ε4 allele [21] and motor deterioration [22], suggesting that lymphocyte reduction has potential predictive power for outcome in PD patients. Taken together, against a background of BBB vulnerability, decreased circulating CD4+ T cells play a crucial role in PD progression, possibly through enhancement of Th1-dependent mechanisms [3,4]. The imbalance in CD4+ T cell transcription factors could be of great interest since it represents a peculiar molecular signature shared by isolated RBD and PD patients as well as potential biomarkers of motor complications [3]. On the other hand, the exact role of B lymphocytes in PD pathogenesis is not fully understood.

In contrast to the lymphocyte count, we were not able to find an association between the neutrophil count and clinical outcome in our study. The role of neutrophils in PD etiopathogenesis has been addressed in several studies with discrepant findings. The baseline neutrophil count could predict PD motor deterioration in the PPMI cohort [11]. A lower lymphocyte count was significantly associated with lower SBR levels on DAT-SPECT, but this association was inconsistent in their original cohort and the PPMI cohort [18]. Notably, the PPMI cohort included early-stage patients who were not receiving dopaminergic treatment. Because neutrophils have dopamine receptors [31], dopaminergic treatment could have modulated neutrophil function in patients who received PD treatment. Another possibility is that neutrophil alterations might not be primarily related to neurodegeneration, but these alterations might play a secondary role and contribute to sustained chronic inflammation through the release of reactive oxygen species, producing a broad spectrum of pro-inflammatory cytokines, neutrophil extracellular traps and alterations in neutrophil-derived proteolytic activity [32]. Further large and longitudinal studies are needed to assess the role of neutrophils in PD.

The present study has several limitations. First, our study lacked a control group. Age-related alterations in the immune system include immunosenescence and an impaired adaptive immune system, and blood cell counts change with age, albeit only slightly [33]. However, we comprehensively evaluated motor and nonmotor symptoms of PD, along with striatal DAT-SPECT and cardiac MIBG scintigraphy uptake, and assessed the disease course according to differences in blood markers between patients. Second, the small sample size and reliance on data from a single center may have limited the generalizability. Third, we performed a comprehensive assessment at baseline but not at the final follow-up. The reason for this is that the purpose of this study was to assess the impact of baseline blood markers on the subsequent clinical course of PD. Fourth, we cannot exclude the possibility of unmeasured or residual confounding factors, such as infections, noncancer conditions, or smoking history, at the time of blood collection. Fifth, information on other inflammatory markers, specifically cytokines, was not available. In the present study, we focused on the use of routine blood cell markers to predict the course of PD, and a more comprehensive study including lymphocyte subtypes and cytokines is needed to understand the effects of peripheral inflammation on PD outcomes.

## 6 Conclusions

We found that a lower lymphocyte count at baseline was associated with a subsequent worse outcomes of PD. However, future studies are necessary to validate the prognostic value of a lower lymphocyte count for PD progression.

## CRediT authorship contribution statement

**Shinsuke Omata:** Writing – review & editing, Project administration, Methodology, Investigation, Data curation, Conceptualization. **Hiroaki Fujita:** Writing – original draft, Project administration, Data curation, Conceptualization. **Hirotaka Sakuramoto:** Writing – review & editing, Methodology, Conceptualization. **Keitaro Ogaki:** Writing – review & editing, Investigation, Conceptualization. **Keisuke Suzuki:** Writing – review & editing, Supervision, Formal analysis, Conceptualization.

## Funding

None.

## Declaration of competing interest

The authors declare the following financial interests/personal relationships which may be considered as potential competing interests: Hiroaki Fujita, Hirotaka Sakuramoto, Keisuke Suzuki reports a relationship with Kyowa Kirin Co Ltd that includes: speaking and lecture fees. Hiroaki Fujita, Hirotaka Sakuramoto, Keisuke Suzuki reports a relationship with Otsuka Pharmaceutical Co Ltd that includes: speaking and lecture fees. Hiroaki Fujita, Hirotaka Sakuramoto, Keisuke Suzuki reports a relationship with Sumitomo Pharma Co Ltd that includes: speaking and lecture fees. Hiroaki Fujita, Hirotaka Sakuramoto, Keisuke Suzuki reports a relationship with Takeda Pharmaceutical Co Ltd Tokyo Headquarters that includes: speaking and lecture fees. Hiroaki Fujita, Hirotaka Sakuramoto, Keisuke Suzuki reports a relationship with Eisai Co Ltd that includes: speaking and lecture fees. Hiroaki Fujita, Hirotaka Sakuramoto, Keisuke Suzuki reports a relationship with Ono Pharmaceutical Co Ltd that includes: speaking and lecture fees. Hiroaki Fujita, Hirotaka Sakuramoto, Keisuke Suzuki reports a relationship with AbbVie Inc that includes: speaking and lecture fees. Hiroaki Fujita, Hirotaka Sakuramoto, Keisuke Suzuki reports a relationship with FP Pharmaceutical Corporation that includes: speaking and lecture fees. Keisuke Suzuki reports a relationship with MSD KK that includes: speaking and lecture fees. If there are other authors, they declare that they have no known competing financial interests or personal relationships that could have appeared to influence the work reported in this paper.

## Appendix A Supplementary data

Supplementary Figure 1 Flowchart of the study.

Supplementary Figure 2 Kaplan-Meier curves for reaching an endpoint by high and low neutrophil count, monocyte count, and NLR.

## References

[1] Cappellano G., Carecchio M., Fleetwood T., Magistrelli L., Cantello R., Dianzani U., Comi C. (2013). Immunity and inflammation in neurodegenerative diseases. Am. J. Neurodegener. Dis..

[2] Tan E.K., Chao Y.X., West A., Chan L.L., Poewe W., Jankovic J. (2020). Parkinson disease and the immune system - associations, mechanisms and therapeutics. Nat. Rev. Neurol..

[3] Contaldi E., Magistrelli L., Comi C. (2022). T Lymphocytes in Parkinson's disease. J. Parkinsons Dis..

[4] Magistrelli L., Storelli E., Rasini E., Contaldi E., Comi C., Cosentino M., Marino F. (2020). Relationship between circulating CD4+ T lymphocytes and cognitive impairment in patients with Parkinson's disease. Brain Behav. Immun..

[5] Kustrimovic N., Comi C., Magistrelli L., Rasini E., Legnaro M., Bombelli R., Aleksic I., Blandini F., Minafra B., Riboldazzi G., Sturchio A., Mauri M., Bono G., Marino F., Cosentino M. (2018). Parkinson's disease patients have a complex phenotypic and functional Th1 bias: cross-sectional studies of CD4+ Th1/Th2/T17 and Treg in drug-naïve and drug-treated patients. J. Neuroinflammation.

[6] Brochard V., Combadière B., Prigent A., Laouar Y., Perrin A., Beray-Berthat V., Bonduelle O., Alvarez-Fischer D., Callebert J., Launay J.M., Duyckaerts C., Flavell R.A., Hirsch E.C., Hunot S. (2009). Infiltration of CD4+ lymphocytes into the brain contributes to neurodegeneration in a mouse model of Parkinson disease. J. Clin. Invest..

[7] Sulzer D., Alcalay R.N., Garretti F., Cote L., Kanter E., Agin-Liebes J., Liong C., McMurtrey C., Hildebrand W.H., Mao X., Dawson V.L., Dawson T.M., Oseroff C., Pham J., Sidney J., Dillon M.B., Carpenter C., Weiskopf D., Phillips E., Mallal S., Peters B., Frazier A., Lindestam Arlehamn C.S., Sette A. (2017). T cells from patients with Parkinson's disease recognize α-synuclein peptides. Nature.

[8] Morris H.R., Spillantini M.G., Sue C.M., Williams-Gray C.H. (2024). The pathogenesis of Parkinson's disease. Lancet.

[9] Yuan X., Wan L., Chen Z., Long Z., Chen D., Liu P., Fu Y., Zhu S., Peng L., Qiu R., Tang B., Jiang H. (2024). Peripheral inflammatory and immune landscape in multiple system atrophy: a cross-sectional study. Mov. Disord..

[10] Muñoz-Delgado L., Macías-García D., Jesús S., Martín-Rodríguez J.F., Labrador-Espinosa M., Jiménez-Jaraba M.V., Adarmes-Gómez A., Carrillo F., Mir P. (2021). Peripheral immune profile and neutrophil-to-lymphocyte ratio in Parkinson's disease. Mov. Disord..

[11] Kim R., Kang N., Byun K., Park K., Jun J.S. (2023). Prognostic significance of peripheral neutrophils and lymphocytes in early untreated Parkinson's disease: an 8-year follow-up study. J. Neurol. Neurosurg. Psychiatry.

[12] Grillo P., Sancesario G.M., Bovenzi R., Zenuni H., Bissacco J., Mascioli D., Simonetta C., Forti P., Degoli G.R., Pieri M., Chiurchiù V., Stefani A., Mercuri N.B., Schirinzi T. (2023). Neutrophil-to-lymphocyte ratio and lymphocyte count reflect alterations in central neurodegeneration-associated proteins and clinical severity in Parkinson Disease patients. Parkinsonism Relat. Disord..

[13] Muñoz-Delgado L., Macías-García D., Periñán M.T., Jesús S., Adarmes-Gómez A.D., Bonilla Toribio M., Buiza Rueda D., Jiménez-Jaraba M.D.V., Benítez Zamora B., Díaz Belloso R., García-Díaz S., Martín-Bórnez M., Pineda Sánchez R., Carrillo F., Gómez-Garre P., Mir P. (2023). Peripheral inflammatory immune response differs among sporadic and familial Parkinson's disease. NPJ Parkinsons. Dis..

[14] Li F., Weng G., Zhou H., Zhang W., Deng B., Luo Y., Tao X., Deng M., Guo H., Zhu S., Wang Q. (2024). The neutrophil-to-lymphocyte ratio, lymphocyte-to-monocyte ratio, and neutrophil-to-high-density-lipoprotein ratio are correlated with the severity of Parkinson's disease. Front. Neurol..

[15] Wang L.X., Liu C., Shao Y.Q., Jin H., Mao C.J., Chen J. (2022). Peripheral blood inflammatory cytokines are associated with rapid eye movement sleep behavior disorder in Parkinson's disease. Neurosci. Lett..

[16] Jensen M.P., Jacobs B.M., Dobson R., Bandres-Ciga S., Blauwendraat C., Schrag A., Noyce A.J. (2021). Lower lymphocyte count is associated with increased risk of parkinson's disease. Ann. Neurol..

[17] Garretti F., Agalliu D., Lindestam Arlehamn C.S., Sette A., Sulzer D. (2019). Autoimmunity in Parkinson's disease: the role of α-synuclein-specific T cells. Front. Immunol..

[18] Muñoz-Delgado L., Labrador-Espinosa M., Macías-García D., Jesús S., Benítez Zamora B., Fernández-Rodríguez P., Adarmes-Gómez A.D., Reina Castillo M.I., Castro-Labrador S., Silva-Rodríguez J., Carrillo F., García Solís D., Grothe M.J., Mir P. (2023). Peripheral inflammation is associated with dopaminergic degeneration in Parkinson's Disease. Mov. Disord..

[19] Williams-Gray C.H., Wijeyekoon R., Yarnall A.J., Lawson R.A., Breen D.P., Evans J.R., Cummins G.A., Duncan G.W., Khoo T.K., Burn D.J., Barker R.A. (2016). Serum immune markers and disease progression in an incident Parkinson's disease cohort (ICICLE-PD). Mov. Disord..

[20] Umehara T., Oka H., Nakahara A., Matsuno H., Murakami H. (2020). Differential leukocyte count is associated with clinical phenotype in Parkinson's disease. J. Neurol. Sci..

[21] Tsukita K., Sakamaki-Tsukita H., Takahashi R. (2021). Lower circulating lymphocyte count predicts apoe ε4-related cognitive decline in Parkinson's disease. Mov. Disord..

[22] McFleder R.L., Musacchio T., Keller J., Knorr S., Petschner T., Chen J.Z., Muthuraman M., Badr M., Harder-Rauschenberger L., Kremer F., Asci S., Steinhauser S., Karl A.K., Brotchie J.M., Koprich J.B., Volkmann J., Ip C.W. (2025). Deep brain stimulation halts Parkinson's disease-related immune dysregulation in the brain and peripheral blood. Brain Behav. Immun..

[23] Postuma R.B., Berg D., Stern M., Poewe W., Olanow C.W., Oertel W., Obeso J., Marek K., Litvan I., Lang A.E., Halliday G., Goetz C.G., Gasser T., Dubois B., Chan P., Bloem B.R., Adler C.H., Deuschl G. (2015). MDS clinical diagnostic criteria for Parkinson's disease. Mov. Disord..

[24] Schade S., Mollenhauer B., Trenkwalder C. (2020). Levodopa equivalent dose conversion factors: an updated proposal including opicapone and safinamide. Mov Disord Clin Pract.

[25] Saito S., Ayabe-Kanamura S., Takashima Y., Gotow N., Naito N., Nozawa T., Mise M., Deguchi Y., Kobayakawa T. (2006). Development of a smell identification test using a novel stick-type odor presentation kit. Chem. Senses.

[26] Miyamoto T., Miyamoto M., Iwanami M., Kobayashi M., Nakamura M., Inoue Y., Ando C., Hirata K. (2009). The REM sleep behavior disorder screening questionnaire: validation study of a Japanese version. Sleep Med..

[27] Matsushima M., Yabe I., Hirotani M., Kano T., Sasaki H. (2014). Reliability of the Japanese version of the scales for outcomes in Parkinson's disease-autonomic questionnaire. Clin. Neurol. Neurosurg..

[28] Fujita H., Suzuki K., Numao A., Watanabe Y., Uchiyama T., Miyamoto T., Miyamoto M., Hirata K. (2016). Usefulness of cardiac MIBG scintigraphy, olfactory testing and substantia nigra hyperechogenicity as additional diagnostic markers for distinguishing between parkinson's disease and atypical parkinsonian syndromes. PLoS One.

[29] Yoshii F., Ryo M., Baba Y., Koide T., Hashimoto J. (2017). Combined use of dopamine transporter imaging (DAT-SPECT) and (123)I-metaiodobenzylguanidine (MIBG) myocardial scintigraphy for diagnosing Parkinson's disease. J. Neurol. Sci..

[30] Dupont W.D., Plummer W.D. (1990). Power and sample size calculations. A review and computer program. Control Clin. Trials.

[31] Sarkar C., Basu B., Chakroborty D., Dasgupta P.S., Basu S. (2010). The immunoregulatory role of dopamine: an update. Brain Behav. Immun..

[32] Rossi B., Constantin G., Zenaro E. (2020). The emerging role of neutrophils in neurodegeneration. Immunobiology.

[33] Tansey M.G., Wallings R.L., Houser M.C., Herrick M.K., Keating C.E., Joers V. (2022). Inflammation and immune dysfunction in Parkinson disease. Nat. Rev. Immunol..

