# Supplementary figures and images for: Prognostic significance of peripheral lymphocyte counts in Parkinson’s disease
Source: Clin Park Relat Disord. 2025 May 10;12:100344. doi: 10.1016/j.prdoa.2025.100344 (PMC12160012; doi:10.1016/j.prdoa.2025.100344)

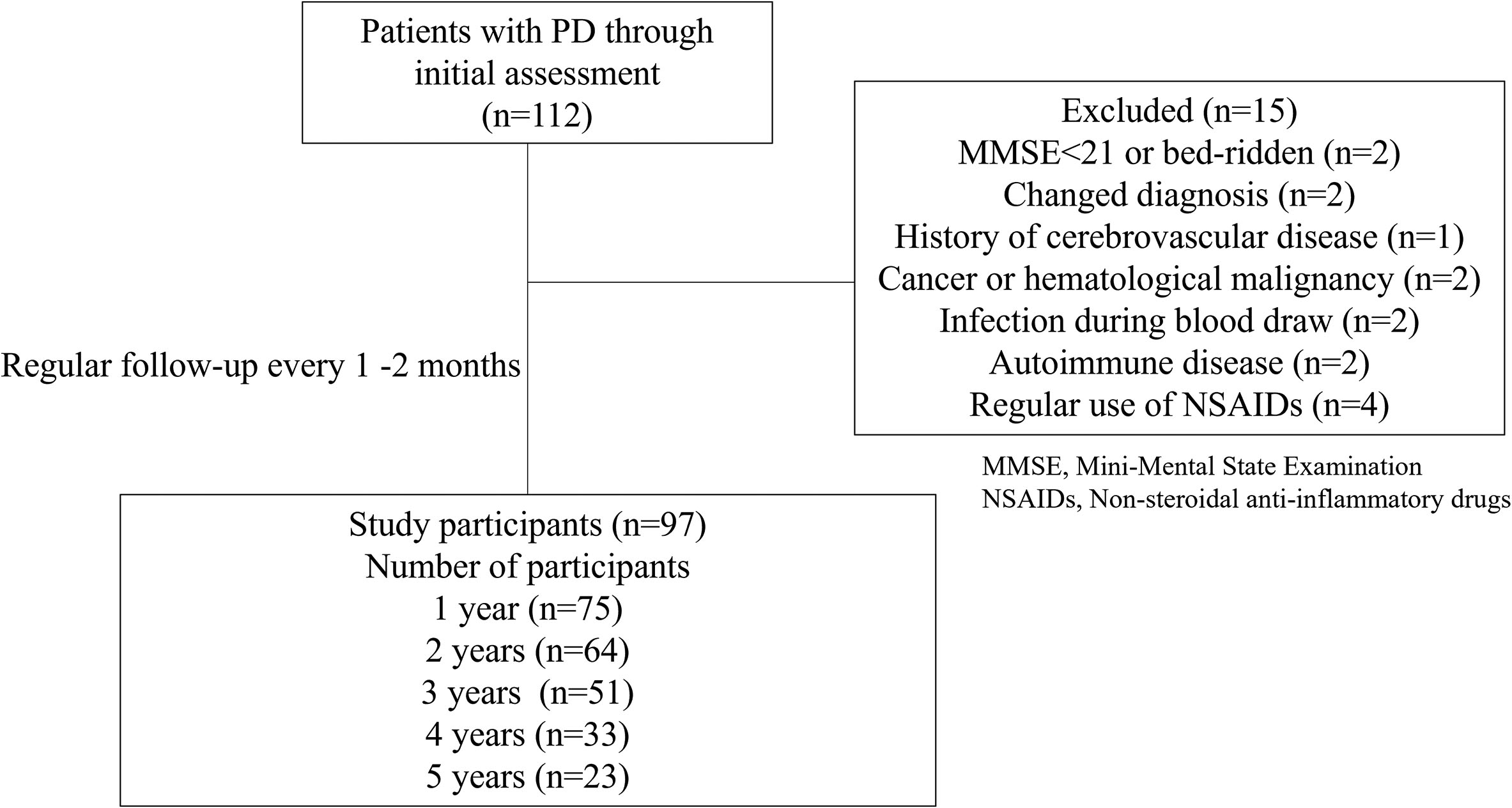

Supplement: Supplementary Figure 1 — Flowchart of the study. [file mmc1.jpg]

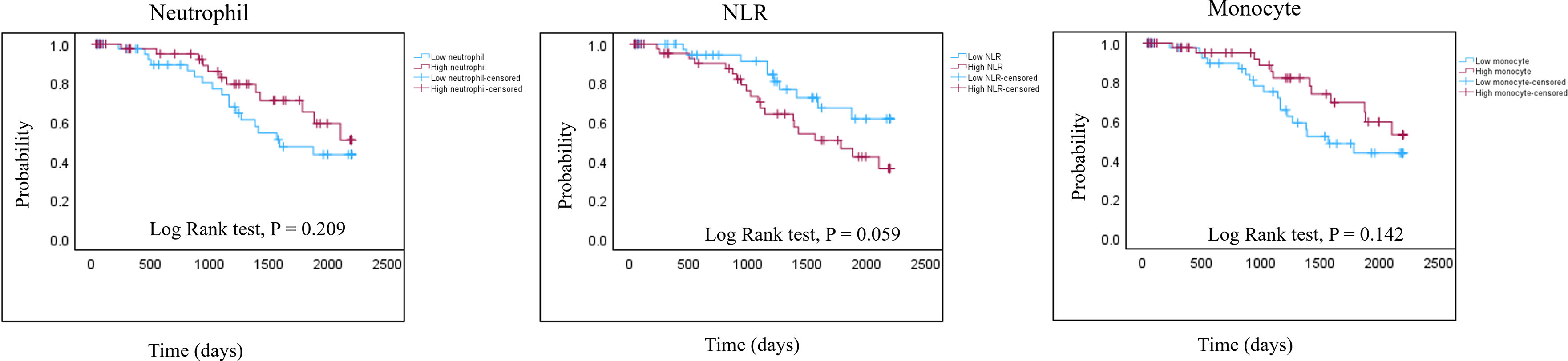

Supplement: Supplementary Figure 2 — Kaplan-Meier curves for reaching an endpoint by high and low neutrophil count, monocyte count, and NLR. [file mmc2.jpg]
