# Supplementary material for: Prognostic significance of peripheral lymphocyte counts in Parkinson’s disease
Source: Clin Park Relat Disord. 2025 May 10;12:100344. doi: 10.1016/j.prdoa.2025.100344 (PMC12160012; doi:10.1016/j.prdoa.2025.100344)
Supplement: Supplementary Data 3 [file mmc3.docx]

**Supplementary Table 1. The clinical backgrounds of all the participants and each group on the basis of the baseline lymphocyte count**

|  | **Low lymphocyte**  **(n=49)** | **High lymphocyte**  **(n=48)** | **P value** |
| --- | --- | --- | --- |
| **Age (years)** | 70.7±9.0 | 67.6±8.2 | **0.039** |
| **Sex (M/F)** | 21/28 | 27/21 | 0.187 |
| **De novo (%)** | 15 (30.6) | 13 (27.1) | 0.701 |
| **PD duration (years)** | 3.6±4.1 | 3.1±2.6 | 0.334 |
| **MMSE score** | 25.0±6.3 | 24.5±7.0 | 0.723 |
| **HY stage** | 2.7±1.1 | 2.5±0.8 | 0.546 |
| **MDS-UPDRS III score** | 31.7±18.2 | 31.7±13.2 | 0.777 |
| **LEDs (mg/day)** | 343.0±347.7 | 306.3±301.3 | 0.787 |
| **SCOPA-AUT** | 11.8±7.9 | 10.0±7.7 | 0.168 |
| **DAT-SPECT SBR (low)** | 3.2±1.1 | 3.2±1.1 | 0.847 |
| **MIBG H/M**  **(early)** | 2.1±0.6 | 2.5±3.7 | 0.506 |
| **MIBG H/M**  **(delay)** | 2.2±3.5 | 1.8±0.8 | 0.306 |
| **Olfactory test score** | 3.4±2.3 | 4.0±2.2 | 0.157 |
| **PDSS-2 score** | 12.9±8.6 | 12.6±9.2 | 0.838 |
| **ESS score** | 8.2±6.4 | 6.8±5.1 | 0.466 |
| **RBDSQ-J score** | 2.5±2.8 | 2.8±5.0 | 0.748 |
| **BDI-II score** | 14.1±8.5 | 12.4±9.2 | 0.275 |
| **Neutrophil count (×10^3^/μL)** | 3.80±0.96 | 3.85±1.02 | 0.937 |
| **Lymphocyte count**  **(×10^3^/μL)** | 1.10±0.25 | 1.87±0.44 | **<0.001** |
| **NLR** | 3.64±1.88 | 2.14±0.80 | **<0.001** |
| **Monocyte count**  **(×10^3^/μL)** | 0.297±0.086 | 0.380±0.132 | **<0.001** |

The data are presented as the n (%) or means ± standard deviations (SDs). Statistically significant differences (P < 0.05) are shown in bold.

PD, Parkinson’s disease; MMSE, Mini-Mental State Examination; HY, Hoehn and Yahr; MDS-UPDRS, Movement Disorder Society Unified Parkinson’s Disease Rating Scale; LEDs, levodopa equivalent doses; SCOPA-AUT, Scales for Outcomes in Parkinson’s Disease–Autonomic; DAT-SPECT, dopamine transporter single-photon emission computed tomography; SBR, specific binding ratio; MIBG, metaiodobenzylguanidine; H/M ratio, heart‒to-mediastinum ratio; NLR, neutrophil‒lymphocyte ratio
